# Prevalence of Temporomandibular Disorders Among Undergraduate Dental Students at the College of Dentistry, Majmaah University, Al‐Zulfi, Saudi Arabia

**DOI:** 10.1155/ijod/6685406

**Published:** 2025-10-31

**Authors:** Khalid A. AL-Hamad

**Affiliations:** ^1^ Department of Oral and Maxillofacial Diagnostic Sciences, College of Dentistry, Qassim University, Buraydah, Saudi Arabia, qu.edu.sa; ^2^ Department of Maxillofacial and Diagnostic Sciences, College of Dentistry, Majmaah University, Al-Majmaah, 11952, Saudi Arabia, mu.edu.sa

**Keywords:** DC/TMD, dental students, preclinical, stress, temporomandibular disorders (TMDs)

## Abstract

**Purpose:**

Temporomandibular disorders (TMDs) are a major cause of nondental pain in the orofacial region. Dental students represent a high‐risk group for developing TMDs due to the considerable academic and clinical stressors of their training. This study aimed to evaluate the prevalence of TMD symptoms and associated factors among dental students at the College of Dentistry, Majmaah University, Saudi Arabia.

**Methods:**

A cross‐sectional study was conducted from April 2023 to September 2024. A self‐administered electronic questionnaire, adapted from established instruments and validated by an expert, was distributed to all undergraduate dental students (*N* = 181). The questionnaire collected demographic data and assessed TMD signs and symptoms using a Likert scale. Data from 165 respondents (response rate: 91.2%) were analyzed using the Mann–Whitney *U* test, with statistical significance set at *p*  < 0.05.

**Results:**

The prevalence of TMD symptoms, based on a defined cutoff score, was 50.3% (83/165). A significantly higher prevalence was observed among clinical‐level students (69.1% of the sample) compared to preclinical students (30.9%; *p*  = 0.019). Female students also reported significantly higher rates of specific symptoms, such as muscular pain during chewing (*p*  = 0.005).

**Conclusion:**

The findings confirm a high prevalence of TMD symptoms among dental students, strongly associated with the advanced clinical phase of training and female gender. We recommend that dental institutions implement educational programs and accessible screening protocols. Students experiencing symptoms, such as pain, clicking sounds, or limited jaw movement should be referred to specialists for early intervention to prevent chronic complications and mitigate the impact on their academic and clinical performance.

## 1. Introduction

Temporomandibular disorders (TMDs) refer to clinical alterations affecting the masticatory muscles, temporomandibular joint (TMJ), and associated structures. TMDs are widespread chronic conditions and are the most common cause of facial pain after toothache, primarily affecting the masseter muscle [1, 2]. These disorders predominantly affect adults in their 20s and 40s [3]. The etiology of TMDs is complex and multifactorial, with psychological factors playing a significant role [4–6]. One of the most debated etiologies is orthodontic treatment, which has been extensively [7], though this association remains controversial. Other contributing factors include jaw injuries, arthritis, long‐term bruxism, and occlusal interferences. Additionally, studies indicate that musculoskeletal disorders are often associated with pain, anxiety, and stress [7].

Diagnosis of TMDs is primarily based on clinical examination and radiographic assessment. One of the most reliable diagnostic tools is the Research Diagnostic Criteria for TMDs (RDC/TMD), introduced by Dworkin and LeResche [8] in 1992. This standardized tool has been widely used due to its accuracy and reliability [9]. It consists of two components: Axis I, which provides a clinical assessment protocol for TMD diagnosis, and Axis II, which evaluates psychological status and pain‐related disability [9]. Another commonly used assessment tool is Fonseca’s Anamnestic Index (FAI) questionnaire, first described by Fonseca et al. [10]. This self‐administered questionnaire consists of 10 questions, is easy to apply, and has demonstrated good accuracy [11, 12].

A study by Srivastava et al. [6], which used RDC/TMD, found that students with TMDs exhibited higher levels of anxiety and parafunctional habits. Furthermore, research has indicated that preclinical students tend to have a lower incidence of TMDs compared to clinical‐level students. Previous studies estimate the prevalence of TMDs among dental students to be approximately 30%–50%. The significance of studying TMDs among dental students lies in their high susceptibility to stress and anxiety due to the demands of clinical practice and academic workload [12].

## 2. Objectives

This study aimed to evaluate the prevalence of TMDs among male and female dental students at the College of Dentistry, Majmaah University, Al‐Zulfi, Saudi Arabia. Additionally, it examined the correlation between psycho‐emotional factors and TMD prevalence among preclinical students (1^st^ and 2^nd^ years) compared to clinical students (3^rd^, 4^th^, and 5^th^ years, as well as interns).

## 3. Materials and Methods

This cross‐sectional study was conducted between April 2023 and September 2024. The study received ethical approval from the Research Ethics Committee of Majmaah University (MUREC) under approval number MUREC‐Mar. 26/COM‐2023/12‐3.

**Figure 1:**
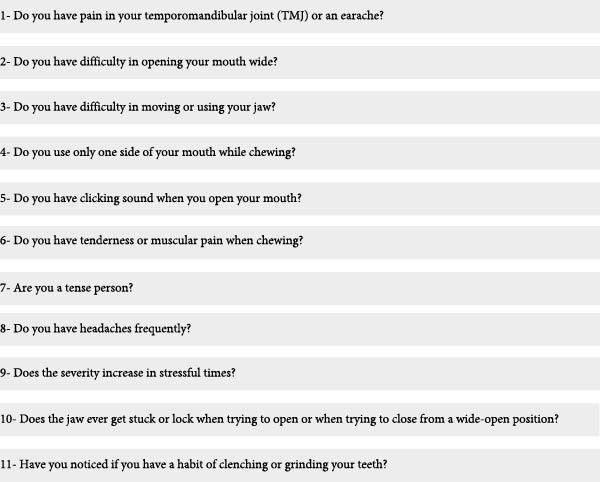
Illustratration of the questions from the second category of the questionnaire.

Data were collected using a self‐administered electronic questionnaire in English, distributed via university email to all undergraduate dental students at the College of Dentistry, Majmaah University. The questionnaire was designed and modified based on previous studies [9, 13]. It consisted of 11 questions rated on a five‐point Likert scale (1 = strongly disagree, 2 = disagree, 3 = neutral, 4 = agree, and 5 = strongly agree), with a total score ranging from 11 to 55 (Figure 1). A cutoff score of ≥28 was used to indicate TMDs. The questionnaire was reviewed and validated by an oral medicine expert. Informed consent was obtained from all participants, and exclusion criteria were verified before proceeding with the questionnaire.

The questionnaire comprised two sections: demographic data (gender, academic level, and GPA) and 11 questions on TMD signs and symptoms.

All dental students (*N* = 181), including interns, were invited to participate. A total of 165 students responded. Students were categorized as preclinical (first and second year, no clinical duties) or clinical (third, fourth, fifth year, and interns, with clinical exposure). GPA was categorized as below 4.0 or equal to/above 4.0.

Exclusion criteria included a history of orthodontic treatment, maxillofacial surgery, jaw trauma, autoimmune disorders (e.g., rheumatoid arthritis), or neurological disorders (e.g., migraine).

Statistical analysis was performed using IBM SPSS Statistics version 22.0. The Shapiro–Wilk test indicated a non‐normal distribution for the Likert‐scale data (*p*  < 0.05); thus, nonparametric tests were used. Descriptive statistics for demographic data are presented as frequencies and percentages. For Likert‐scale data, medians are reported as the primary measure of central tendency, with means and standard deviations provided for supplementary context, along with frequencies and percentages for each response category (Table 1).

**Table 1:** Collective responses of participants regarding TMJ Pain.

S. no.	Question	Median (50^th^ percentile)^a^	Mean ± SD	Highly disagree		Disagree		Neutral		Agree		Highly agree	
				*N*	(%)	*N*	(%)	*N*	(%)	*N*	(%)	*N*	(%)
1	Do you have pain in your temporomandibular joint (TMJ) or an earache?	3.00	2.62 ± 1.29	45	27.3	37	22.4	26	15.8	49	29.7	8	4.8
2	Do you have difficulty opening your mouth wide?	2.00	2.58 ± 1.29	48	29.1	35	21.2	27	16.4	48	29.1	7	4.2
3	Do you have difficulty moving or using your jaw?	2.00	2.35 ± 1.18	50	30.3	47	28.5	34	20.6	28	17.0	6	3.6
4	Do you use only one side of your mouth while chewing?	3.00	2.62 ± 1.27	46	27.9	33	20.0	27	16.4	55	33.3	4	2.4
5	Do you hear a clicking sound when you open your mouth?	4.00	3.15 ± 1.34	31	18.8	25	15.2	16	9.7	74	44.8	19	11.5
6	Do you have tenderness or muscular pain when chewing?	2.00	2.34 ± 1.23	58	35.2	39	23.6	24	14.5	42	25.5	2	1.2
7	Are you a tense person?	3.00	3.15 ± 1.10	16	9.7	30	18.2	43	26.1	65	39.4	11	6.7
8	Do you frequently experience headaches?	3.00	3.13 ± 1.15	20	12.1	29	17.6	35	21.2	71	43.0	10	6.1
9	Does the severity of your symptoms increase during stressful times?	3.00	3.10 ± 1.24	24	14.5	30	18.2	34	20.6	60	36.4	17	10.3
10	Does your jaw ever get stuck or lock when trying to open or close from a wide‐open position?	2.00	2.05 ± 1.12	67	40.6	52	31.5	18	10.9	26	15.8	2	1.2
11	Have you noticed a habit of clenching or grinding your teeth?	3.00	2.74 ± 1.27	35	21.2	44	26.7	26	15.8	49	29.7	11	6.7

Abbreviation: SD, Standard‐deviation.

^a^The median is the middle value in a set of numbers and is equivalent to the 50^th^ percentile.

The Mann–Whitney *U* test was used to compare responses between independent subgroups (gender, academic level, and GPA), with results presented as mean ranks and *p*‐values (Table 2). Spearman’s rank correlation coefficient (*ρ*) was used to assess monotonic relationships between all questions, with the correlation matrix presented in Table 3. Statistical significance was set at *p*  < 0.05 for all tests.

**Table 2:** Comparison of responses between gender, academic level, and GPA.

S. no.	Questions	Gender			Academic level			Grade point average (GPA)		
		Male mean rank (*N* = 74)	Female mean rank (*N* = 91)	*p*‐Value	Preclinical (1^st^ year and 2^nd^ year) (*N* = 51)	Clinical (3^rd^, 4^th^, 5^th^ year, and interns) (*N* = 114)	*p*‐Value	Below 4 (*N* = 26)	Above 4 (*N* = 139)	*p*‐Value
1	Do you have pain in your temporomandibular joint (TMJ) or an earache?	76.36	88.40	0.097	70.34	88.66	**0.019**	85.81	82.47	0.736
2	Do you have difficulty opening your mouth wide?	81.26	84.42	0.662	76.53	85.89	0.229	87.44	82.17	0.594
3	Do you have difficulty moving or using your jaw?	76.67	88.15	0.112	72.31	87.78	**0.047**	80.62	83.45	0.774
4	Do you use only one side of your mouth while chewing?	87.72	79.16	0.235	77.79	85.33	0.331	93.21	81.09	0.218
5	Do you hear a clicking sound when you open your mouth?	84.09	82.12	0.781	73.48	87.26	0.071	89.12	81.86	0.453
6	Do you have tenderness or muscular pain when chewing?	71.77	92.13	**0.005**	70.59	88.55	**0.020**	94.65	80.82	0.159
7	Are you a tense person?	83.79	82.36	0.841	71.69	88.06	**0.033**	83.15	82.97	0.985
8	Do you frequently experience headaches?	81.45	84.26	0.693	70.49	88.60	**0.018**	84.62	82.70	0.843
9	Does the severity of your symptoms increase during stressful times?	82.14	83.70	0.828	76.26	86.01	0.210	90.42	81.61	0.372
10	Does your jaw ever get stuck or lock when trying to open or close from a wide‐open position?	78.91	86.33	0.294	81.26	83.78	0.742	83.77	82.86	0.925
11	Have you noticed a habit of clenching or grinding your teeth?	79.85	85.56	0.431	74.54	86.79	0.117	97.62	80.27	0.080

*Note:* Values in bold indicate statistically significant results (*p* < 0.05).

**Table 3:** Correlation between responses.

Questions	Do you have temporomandibular joint (TMJ) pain or earache?	Do you have difficulty opening your mouth wide?	Do you have difficulty moving or using your jaw?	Do you use only one side of your mouth while chewing?	Do you hear a clicking sound when you open your mouth?	Do you have tenderness or muscular pain when chewing?	Are you a tense person?	Do you frequently experience headaches?	Does the severity of your symptoms increase during stressful times?	Does your jaw ever get stuck or lock when trying to open or close from a wide‐open position?	Have you noticed a habit of clenching or grinding your teeth?
Do you have pain in your temporomandibular joint (TMJ) or an earache?	1	**0.620**	**0.644**	**0.314**	**0.416**	**0.597**	**0.254**	**0.210**	**0.182**	**0.329**	**0.336**
	—	**0.000**	**0.000**	**0.000**	**0.000**	**0.000**	**0.001**	**0.007**	**0.019**	**0.000**	**0.000**
Do you have difficulty opening your mouth wide?	**0.620**	1	**0.703**	**0.349**	**0.395**	**0.522**	**0.203**	**0.206**	**0.185**	**0.323**	**0.182**
	**0.000**	—	**0.000**	**0.000**	**0.000**	**0.000**	**0.009**	**0.008**	**0.017**	**0.000**	**0.020**
Do you have difficulty moving or using your jaw?	**0.644**	**0.703**	1	**0.421**	**0.389**	**0.574**	**0.305**	**0.234**	0.151	**0.357**	**0.280**
	**0.000**	**0.000**	—	**0.000**	**0.000**	**0.000**	**0.000**	**0.002**	0.053	**0.000**	**0.000**
Do you use only one side of your mouth while chewing?	**0.314**	**0.349**	**0.421**	1	**0.424**	**0.417**	**0.246**	0.135	0.132	**0.267**	**0.169**
	**0.000**	**0.000**	**0.000**	—	**0.000**	**0.000**	**0.001**	0.085	0.092	**0.001**	**0.030**
Do you hear a clicking sound when you open your mouth?	**0.416**	**0.395**	**0.389**	**0.424**	1	**0.367**	**0.351**	**0.303**	**0.259**	**0.237**	**0.348**
	**0.000**	**0.000**	**0.000**	**0.000**	—	**0.000**	**0.000**	**0.000**	**0.001**	**0.002**	**0.000**
Do you have tenderness or muscular pain when chewing?	**0.597**	**0.522**	**0.574**	**0.417**	**0.367**	1	**0.330**	**0.213**	0.146	**0.269**	**0.193**
	**0.000**	**0.000**	**0.000**	**0.000**	**0.000**	—	**0.000**	**0.006**	0.062	**0.000**	**0.013**
Are you a tense person?	**0.254**	**0.203**	**0.305**	**0.246**	**0.351**	**0.330**	1	**0.580**	**0.475**	0.121	**0.411**
	**0.001**	**0.009**	**0.000**	**0.001**	**0.000**	**0.000**	—	**0.000**	**0.000**	0.120	**0.000**
Do you frequently experience headaches?	**0.210**	**0.206**	**0.234**	**0.135**	**0.303**	**0.213**	**0.580**	1	**0.704**	0.193	**0.315**
	**0.007**	**0.008**	**0.002**	**0.085**	**0.000**	**0.006**	**0.000**	—	**0.000**	0.013	**0.000**
Does the severity of your symptoms increase during stressful times?	**0.182**	**0.185**	0.151	**0.132**	**0.259**	0.146	**0.475**	**0.704**	**1**	**0.224**	**0.333**
	**0.019**	**0.017**	0.053	**0.092**	**0.001**	0.062	**0.000**	**0.000**	**-**	**0.004**	**0.000**
Does your jaw ever get stuck, or lock when trying to open or close from a wide‐open position?	**0.329**	**0.323**	**0.357**	**0.267**	**0.237**	**0.269**	0.121	**0.193**	**0.224**	**1**	**0.296**
	**0.000**	**0.000**	**0.000**	**0.001**	**0.002**	**0.000**	0.120	**0.013**	**0.004**	**-**	**0.000**
Have you noticed a habit of clenching or grinding your teeth?	**0.336**	**0.182**	**0.280**	**0.169**	**0.348**	**0.193**	**0.411**	**0.315**	**0.333**	**0.296**	1
	**0.000**	**0.020**	**0.000**	**0.030**	**0.000**	**0.013**	**0.000**	**0.000**	**0.000**	**0.000**	—

*Note:* All bold values correspond to statistically significant correlations at *p* < 0.05. No non‐significant values are in bold.

## 4. Results

A total of 165 undergraduate dental students participated in this study, yielding a response rate of 91.2%. The demographic and academic characteristics of the participants are presented in Table 4. Of these, 91 (55.2%) were female, and 74 (44.8%) were male. Among the participants, 51 (30.9%) were preclinical students (1^st^ and 2^nd^ year), and 114 (69.1%) were clinical students (3^rd^, 4^th^, and 5^th^ year, including interns).

**Table 4:** Demographic and academic characteristics of the respondents.

S. no.	Variables	Subgroup	Frequency (*N*)	Percentage (%)
1	Gender	Female	91	55.2
		Male	74	44.8

2	Academic year	Preclinical (1^st^ year and 2^nd^ year)	51	30.9
		Clinical (3^rd^, 4^th^, 5^th^ year, and interns)	114	69.1

3	GPA	Below 4	26	15.8
		Equal or above 4	139	84.2

4	History of TMJ disorders	Yes	15	9.1
		No	150	90.9

Abbreviations: GPA, grade point average; TMJ, temporomandibular joint.

When asked about their history of TMJ disorders, 15 (9.1%) participants reported a positive history. However, based on the questionnaire’s cutoff score (≥28), the prevalence of TMD symptoms was 50.3% (83 out of 165). The distribution of responses to each question is detailed in Table 1. Regarding specific symptoms, 34.5% of respondents reported experiencing TMJ pain or earache (responses “Agree” and “Strongly Agree”). Clicking sounds were the most frequently reported symptom, with 56.3% of participants acknowledging their occurrence. In contrast, tenderness or muscle pain during chewing was reported by 26.7% of participants.

Table 2 presents the comparisons of responses based on gender, academic level, and GPA using the Mann–Whitney *U* test. No significant differences were observed in responses for any questions when compared by GPA (*p*  > 0.05 for all questions). In gender‐based comparisons, a significant difference was found only for question 6 ("Do you have tenderness or muscular pain when chewing?"), with females reporting significantly higher scores (*p* = 0.005). Significant differences were observed between preclinical and clinical students for questions 1, 3, 6, 7, and 8 (*p* = 0.019; 0.047, 0.020, 0.033, and 0.018, respectively), with clinical students consistently reporting higher scores.

The Spearman’s correlation matrix between all pairs of questions is presented in Table 3. Several strong and statistically significant positive correlations were observed. Notably, the strongest correlations were found between question 8 (“Do you frequently experience headaches?”) and question 9 (“Does the severity of your symptoms increase during stressful times?”) (*ρ* = 0.704 and *p*  < 0.001), and between question 7 (“Are you a tense person?”) and question 8 (*ρ* = 0.580 and *p*  < 0.001).

## 5. Discussion

TMDs are complex conditions involving structural, functional, or physiological changes in the masticatory system, often leading to orofacial discomfort and associated with various systemic health concerns [13]. As the orofacial region is critical for essential functions like chewing and speaking, TMDs can significantly impact quality of life, with pain being the primary driver for seeking clinical care [14, 15].

This study assessed the prevalence and associated factors of TMDs among undergraduate dental students at Majmaah University using a customized survey adapted from previous instruments, including Fonseca’s questionnaire [6, 16–18]. The observed correlation between TMDs and dental students is well‐documented and often attributed to the heightened stress and anxiety associated with managing demanding academic and clinical responsibilities [19–22]. Other factors, such as smoking, have also been identified as contributors to TMD severity in this population [23].

Our findings align with this premise, demonstrating that clinical‐level students reported significantly higher TMD tendencies than their preclinical counterparts. This disparity is likely due to the increased curricular and practical demands of the clinical years, a pattern consistent with findings by Alamri et al. [21] and Zafar et al. [24]. Furthermore, our results reinforce the established consensus that dental students exhibit a higher prevalence of TMDs compared to other student populations [21, 24].

In this study, 9.1% of participants reported a history of joint disorders, a figure that aligns closely with the 8%–12% prevalence estimate reported by the National Institute of Dental and Craniofacial Research (NIH) [25]. However, based on the defined cutoff score (≥28), the prevalence of TMD symptoms was 50.3%. This discrepancy with the self‐reported history and the wide range of prevalence rates found in other literature (46%–80%) underscores the significant impact of differing methodological approaches and diagnostic criteria across studies [26, 27]. It is also noteworthy that less than 10% of individuals with TMD symptoms seek professional intervention, suggesting a high threshold for perceiving the condition as severe [28].

A key finding was that 34.5% of participants reported TMJ pain or earache. This aligns with studies, such as Rehman et al. [29], who reported a 36.3% prevalence of mild TMD among dental students in Karachi. Furthermore, nearly half of the participants acknowledged that their symptom severity increased during stressful periods, a finding corroborated by Gaş et al. [30], who identified a similar link between TMD and stress during the COVID‐19 pandemic among Turkish dental students.

Consistent with the extensive body of literature [31, 32], our analysis revealed a gender disparity, with female students reporting significantly higher rates of tenderness or muscular pain during chewing than males (*p* = 0.005). This heightened susceptibility among females is often attributed to a combination of hormonal and psychosocial factors, though further research is warranted to fully elucidate this relationship.

Similarly, clinical‐level students reported significantly more TMJ pain and earaches than preclinical students (*p* = 0.019). This finding supports the results of Srivastava et al. [6, 33], who found clinical students had 65% higher odds of having TMD (OR = 1.65), although it contrasts with other studies that found no significant correlation. This inconsistency highlights the need for further investigation into the specific academic stressors impacting TMD development.

This study has several limitations. Its cross‐sectional design precludes the establishment of causal relationships between observed variables. The reliance on a self‐reported questionnaire, albeit validated, is another limitation. Future research should employ longitudinal cohort studies across diverse academic institutions and incorporate standardized clinical examinations, such as the Diagnostic Criteria for TMDs (DC/TMD) encompassing both Axis I and II, to obtain a more comprehensive and objective assessment of TMD prevalence and its psychosocial impact.

## 6. Conclusion

This study revealed a high self‐reported prevalence of TMD symptoms among dental students at Majmaah University, particularly affecting those at the clinical academic level and female students. The significant association between TMD symptoms and academic stress highlights the substantial impact of the demanding dental curriculum on students’ well‐being.

Based on these findings, it is recommended that dental educational institutions implement routine screening programs for TMDs. Students, especially those experiencing pain, joint sounds, or limited jaw movement, should be encouraged to undergo a thorough examination by TMJ specialists. Early identification and management are crucial not only for alleviating student discomfort but also for preventing potential long‐term complications that could adversely affect their academic performance, clinical training, and future careers.

Future longitudinal studies incorporating clinical diagnoses using standardized criteria like the DC/TMD are essential to validate these findings and further explore the complex interplay between academic stress, psychological factors, and TMD development in this population.

## Conflicts of Interest

The author declares no conflicts of interest.

## Funding

No funding was received for this manuscript.

## Data Availability

The data that support the findings of this study are available from the corresponding author upon reasonable request.
